# A Rare Human Helminth Infection in Russia

**DOI:** 10.3390/tropicalmed8080403

**Published:** 2023-08-08

**Authors:** Anatoly V. Kondrashin, Lola F. Morozova, Ekaterina V. Stepanova, Natalia A. Turbabina, Maria S. Maksimova, Alina S. Anikina, Ariyo Shahin-jafari, Aleksandr E. Morozov, Dmitry V. Mikhaylov, Yulia D. Kupriyanova, Evgeny N. Morozov

**Affiliations:** Department of Medical Parasitology and Virology, Sechenov University, Malaya Pirogovskaya Str., 20, 119435 Moscow, Russia; anakona@mail.ru (A.V.K.); lfmorozova@mail.ru (L.F.M.); stepan-kate83@mail.ru (E.V.S.); n.turbabina@mail.ru (N.A.T.); maksimovmarij@yandex.ru (M.S.M.); alianikina19@gmail.com (A.S.A.); ariyo.shahin@gmail.com (A.S.-j.); a.e.morozov@mail.ru (A.E.M.); dima_mixal@mail.ru (D.V.M.); yukup45@mail.ru (Y.D.K.)

**Keywords:** helminthiasis, strongyloidosis, sparganosis, dipylidiosis, fasciolosis, dioctophymosis, capillariosis

## Abstract

Currently, more than 500,000 cases of various helminthes in humans are reported annually in the Russian Federation. This figure may not reflect the true incidence of helminthes, as only nine separate nosological forms are compulsory notifiable. The rest of the species of detected helminthes are included in a separate category of “other helminthes” or “rare helminthes”. The bulk of the latter is represented by the helminthes with a rate of incidence that does not exceed one case per 100,000 people. This review is based on data derived from publications in the Russian language, both from the Russian Federation and international, as well as data available from various health treatment facilities in Russia. These data largely cover the period of the 1990s–2010s. A total of 15 species of “rare helminthes” are described in this review: anisakiosis, capillariosis, clonorchosis, dioctophymosis, dipylidiosis, echinochasmosis, fasciolosis, gastrodiscoidosis (amphistomiosis), metagonimosis, metorchiosis, nanophyetosis, pseudamphistomosis, sparganosis (spirometrosis), strongyloidosis and trichostrongylosis. Details of their geographical distribution, clinical and epidemiological peculiarities, and the difficulties they pose in diagnosis are provided. The public health importance of “rare helminthes” in Russia at present and in the forthcoming years is stressed.

## 1. Introduction

Ever-changing social, economic, and environmental conditions facilitate the evolution of epidemiological processes for many infectious and parasitic diseases across the world. Out of a total of 270 diseases caused by various helminthes worldwide, approximately 70 species have been found in Russia and in areas of the former USSR. Expert assessments of the magnitude of incidence of helminthes in the USSR show that a total of approximately 44 million cases can occur annually, which exceeds the officially recorded incidence by more than 10 times [1].

Following the collapse of the USSR in 1991, more than 500,000 cases of various helminthes have been reported annually in the Russian Federation [2,3]. Evidence exists to show that this figure may be even higher due to inadequate reporting systems.

From 1971 to 1990, official statistics from the former USSR reported only nine separate nosological entities (forms) of helminthes: ascaridosis, trichocephaliosis, taeniosis, taeniarhynchosis, diphyllobothriosis, opisthorchosis, hymenolepidiosis, enterobiosis and ankylostomiosis [3]. From 1991 onwards, this number increased from 9 to 11 through the inclusion of echinococcosis, trichinellosis and toxocariosis, while ankylostomiosis was excluded from the list [3]. The epidemiological profiles of the helminthes forms to be reported are shown in Appendix A.

The rest of the detected helminthes constitute a separate category—“other helminthes”. This consists of worms, the incidence of which did not exceed one case per 100 000 people. The helminthes in this group could be locally endemic but were considered as “rare helminthes” (RH) [2]. A total of 15 species of helminthes in this category were detected in various parts of the country [3].

## 2. Materials and Methods

The objective of this research is to reiterate the need to improve detection, surveillance, and the awareness of health services personnel with respect to “rare helminthes”. The findings of the study may prove to be useful for better understanding the proliferation of the RH forms in the large territory of the Russian Federation. The geographical confinement of the RH may further aid in achieving this objective.

The selection criterion used for the publications was the utilization of available data only on the RH in humans from various sources, like data published in Russian by research institutions, health ministry reports, and various health treatment facilities. Data on the incidence of RH among animals were excluded. For comparative purposes, relevant publications in English were also scrutinized and included in the list of references. Another criterion used for selection was the inclusion of publications with identified helminthes species verified in reference laboratories.

## 3. Results

### 3.1. Distribution of the RH in the Russian Federation

The consolidated data on the distribution of the RH forms in the Russian Federation are presented in Appendix B. Details on the incidence of RH, in terms of federal districts, are provided as follows:

As of 2013, there were total of seven federal okrugs (federal districts) in the territory of the Russian Federation:

- Central Federal District (center—Moscow city), population 38,819,900;
- Northwestern Federal District (center—Saint-Petersburg city), population 13,800,700;
- Southern Federal District (center—Rostov-on-Don city), population 23,554,000;
- Volga River Basin Federal District (center—Nizhni Novgorod city), population 29,738,800;
- Ural Federal District (center—Ekaterinburg city), population 12,234,200;
- Siberian Federal District (center—Novosibirsk city), population 19,292,700;
- Far East Federal District (center—Khabarovsk city), population 6,226,600.

The territorial distribution of RH, along with their epidemiological profiles, are described in the text presented below, as well as Appendix A and Appendix B.

#### 3.1.1. Central Federal District (center—Moscow city)

A few sporadic cases of dipylidiosis (*Dipylidium caninum* L., 1758; *Railliet*, 1892) were reported in the Samara and Tula regions [4].

Several cases of sparganosis (spirometrosis) caused by *Spirometra erinacei europaei* (*Muller*, 1937) were registered in the Tver and Moscow regions [4,5].

A small-scale outbreak of strongyloidosis caused by *Strongyloides stercoralis* (Bavay, 1876; Styles et Hassal, 1902) occurred in Moscow city during the end of the 1990s, and sporadic cases were detected in the following years in the Smolensk region.

A few cases of fasciolosis caused by *Fasciola hepatica* (L., 1758) were registered in the Yaroslavl and Bryansk regions.

During 2007–2015, complex parasitological and epidemiological investigations aimed at detecting helminthes among vertebrates were carried out in the Kursk region of the Central Federal District. A total of 13 species of various helminthes in different animals, both domestic and wild, were detected, and all of them could potentially be causative agents of RH. The detected species were *Alaria alata, Plagiorchis elegans, Dicrocoelium lanceatum, Hydatigera taeniaformis, Taenia martis, Taeniidae* spp., *Dypylidium caninum, Spirometra ernacei*, *Eucoleus aerophilius, Metastrongylus apri*, *Uncinaria stenocephala, Syphacia obvelata,* and *Toxocara canis* [6,7,8].

#### 3.1.2. Northwestern Federal District (Center—Saint-Petersburg)

Cases of sparganosis (*Spirometra erinaceid*, Muller, 1937) were described in the Novgorod region [5].

Cases of dioctophymosis caused by the nematode *Dioctophyme renale (Goeze,* 1782; *Stiles*, 1901) were reported in the Karelia, Leningrad, and Archangelsk regions [4].

Sporadic cases of strongyloidosis were reported in Saint Petersburg city [9].

#### 3.1.3. Southern Federal District (Center—Rostov-on-Don)

There was a great difference between the wide spread of zoonotic fasciolosis in sheep and cattle, with only sporadic cases in humans in Russia [4,10,11].

Sporadic cases of strongyloidosis were registered in the territories of the Dagestan Republic and Krasnodar region [4].

Pseudamphistomosisis is caused by *Pseudamphistomum truncatum (Dudolphi*, 1819; *Luhe*, 1908) from the family *Opistorchidae*. Cases of infection were reported in the basin of the Don River [4].

#### 3.1.4. Volga River Basin Federal District (Center—Nizhni Novgorod)

A total of nine species of RH were reported in this district: capillariosis, dipylidiosis, echinochasmosis, gastrodiscoidosis, fasciolosis, pseudamphistomosis, sparganosis, trichostrongylosis, and strongyloidosis.

Capillariosis is a helminthiasis that is caused by the nematodes *Capillaria hepatica* and *Capillaria philippinensis*. There are several clinical forms of the disease: intestinal, pulmonary, liver, and others [12]. Thus far, only one case of the cutaneous form of infection has been described in the territory of the Astrakhan region [13].

Sporadic cases of dipylidiosis in humans were reported in the Samara region [4].

Echinochasmosisis is an infection caused by the tiny trematode *Echinochasmus perfoliatus* (*Ratz,* 1908; *Dietz*, 1909). Cases of echinochasmosis were registered in the basin of the Volga River, particularly in the Astrakhan and Volgograd regions [4].

Gastrodiscoidosis is caused by the trematode *Gastrodiscoides hominis* (*Lewis et McConnall*, 1876). Currently, sporadic cases of the disease in humans are being reported in the delta of the Volga River and in the Astrakhan and Krasnodar regions [14].

Sporadic cases of fasciolosis in humans were detected in the Samara region [11].

Cases of pseudamphistomosis were registered in the Republic of Tatarstan in 1989–1994. In Kazan, the capital of Tatarstan, several mixed cases with opisthorchosis were found [15].

A few cases of sparganosis were reported in the Astrakhan region in humans [5].

Sporadic cases of trichostrongylosis transmitted by *Trichostrongylus colubriformis* (*Giles*, 1892; *Ranson*, 1911) were reported in the territory of the Astrakhan region [4].

One case of strongyloidosis was detected in Volgograd city [16].

#### 3.1.5. Ural Federal District (Center—Ekaterinbourg)

Cases of RH in humans have not been reported thus far. However, there is a known focus of linguatulosis among cattle in the territory of Orenbourg region, potentially posing a threat to local inhabitants [17,18].

Siberian Federal District (center—Novosibirsk).

Cases of metorchiosis, pseudamphistomosis, and fasciolosis have been reported in this district.

Metorchiosis transmitted by *Metorchis billis* (Braun, 1890) was reported in the territory of Western Siberia, particularly in the Novosibirsk region and in the basin of the Ob’ River [19]. A serological survey of 37 patients carried out in 2000 revealed that 48.7% were positive for *Opisthorchis felineus*, 37.8% showed mixed cases of *Opisthorchis felineus*, and *Methorchis billis*, and 13.5% were positive for *Methorchis billis* [20,21]. There was one report indicating that helminthes were found in the biliary ducts of autopsied humans [22].

Several cases of pseudamphistomosis in humans were reported in Tomsk city in 1989–1994 [23].

Sporadic cases of fasciolosis in humans have been registered in the Yamal-Nenetz autonomous district, the Khanti-Mansi autonomous district, the Republic of Khakassia, Krasnoyarsk Territory, and the Republic of Altai [23].

#### 3.1.6. Far East Federal District (Center—Khabarovsk)

This is the largest federal district in the Russian Federation in terms of both land and incidences of RH. It embraces vast territories of the Prymorye (Maritime) Territory, Khabarovsk Territory, Kamchatka Territory, and a few other administrative entities.

Clonorchosis (*Clonorchis sinensis* (Cobbold, 1875; *Looss*, 1907)) is a prevailing species among other RH in the territory of this federal district, and it annually accounts for 40–50 cases of disease on average [24,25,26]. Cases of clonorchosis have been reported in Khabarovsk city and in the districts of Nanaisk, Amursk, and Komsomolsk. The Amursky region contributes approximately 75% of the total number of cases of the infection [3].

Cases of clonorchosis are more frequent among local people than among migrants; a few lethal cases were reported in association with other diseases [27].

The trematode *Nanophyetus schichobalowi* (*Skrjabin et Podjapolskaya*, 1931), causing nanophyetosis, was described in the ex-USSR in 1928, firstly among the Udugei tribe in the low part of the Amur River basin. An examination of the population of the Amursky region during the 1990s revealed the persistence of this disease’s foci [4,28].

At present, the natural foci of the infection are largely confined to the mountain tributaries of the Amur and Ussuri Rivers [29].

A focus of metagonimosis, caused by *Metagonimus yokogawi* (*Katsurada*, 1912) with a rather high prevalence of infection (66.7%), was found among the original residents of the low part of the Amur River basin [30]. Several cases of metagonimosis were reported among the population of Sakhalin Island and in the territory of the Jewish Autonomous Region [30,31]. A few cases of strongyloidosis were reported in the city of Khabarovsk [4].

The first case of anisakiosis was described in the Kamchatka territory at the beginning of the 2000s. In the following years, sporadic cases of anisakiosis were reported in the populations of a few districts of Sakhalin Island [31,32].

Several cases of metagonimosis were reported among the populations of Sakhalin Island and in the territory of the Jewish Autonomous Region [33].

### 3.2. Target Population

The target population refers to humans only.

There are a number of various social-economic factors, which determine the dis-tribution of geo- and bio-helminthes on the concrete territory. These factors are as fol-lows: sanitary-hygienic habits of local population, sanitary status of territory, occupa-tional activities of people, living standard of people, and alike. Among the habits of people the most important are: absence of latrine, use of any place in the vegetable garden for defecation, use of domestic animals shelters, stables for defecation, use of human feces as a manure, consumption of undercooked/raw fish, crabs and other sea- and fresh water products, use of unfiltered water for drinking and washing vegeta-bles/fruits etc., occupational activities, particularly related to agriculture such as cattle breeding, work at the slaughter house, and alike.

### 3.3. Clinical and Epidemiological Peculiarities of RH in the Russian Federation

There is a marked difference in the spatial distribution and species composition of the RH in the Russian Federation. From an epidemiological point of view, three groups of RH in humans can be distinguished, as described below.

#### 3.3.1. Helminthes Acquired through the Consumption of Infected Fishes and Other Sea/River Products

The available data indicate that representatives of this group contribute to approximately 81% of the total RH cases in the territory of the Russian Federation [3]. They are largely confined to the vast territory of the Far East Federal District: Primorsky, the Khabarovsk and Kamchatka territories, the Amur and Sakhalin regions, and the Jewish Autonomous Region [3].

The major sources of infection for this group include various species of fishes (66.0%) [3]. There is a well-defined trend between the level of fish consumed by the local population and the incidence of RH. Thus, the highest level of fish products consumed is in the Far East Federal District (30.0 kg/person/year), which accounts for 70% of the country’s total fish yield, compared with some other places like the Republic of Tuva, with 7.8 kg/person/year (Siberian Federal District) [31].

Approximately 1000 cases of clonorchosis, metagonimosis, anisakiosis, nanophyetosis, sparganosis, paragonimosis, and dioctophymosis were detected during the last decades in the territory in the far east of Russia.

Clonorchosis is a leading RH in this group. More than 75% of the total cases of clonorchosis were registered in the territory of the Amur region, particularly in areas bordering China. In the Khabarovsk territory, more than 59% of infected persons were found among indigenous people, the Nanai, for whom fish and sea products are staple foods. Adults constituted the majority of infected persons (90.4%). The cases were detected mainly among workers, sales representatives, and office workers (a total of 60.0) [3].

#### 3.3.2. Helminthes Acquired through Contaminated Soil, Water, and Vegetation

Although this group contributed only 17% of the total RH detected in Russian Federation, species of this group were reported in the territories of five out of a total of seven federal okrugs (the Central, North-Western, Southern, Ural, and Siberian Federal Okrugs).

The representatives of this group are fasciolosis, strongyloidosis, and trichostrongylosis. Cases of the former were largely detected in the Krasnodar territory, Republic of Adigeya (Southern Federal District). Approximately 44% of the total cases were detected amongst persons engaged in agriculture-related activities like vegetable growing, horticulture, cattle rearing, etc., thus facilitating contact with contaminated soil. Almost 30% of the total cases were detected in children due to their presumably low levels of hygiene [3].

#### 3.3.3. RH Acquired Due to Contact with Infected Animals

This group consisted of cases of dipylidiosis alone and contributed 2% of the total RH cases. It did not show any particular geographical preponderance and was detected both in rural and urban areas among adults and children in equal proportions in the Central and Volga River Basin Federal Districts [3].

#### 3.3.4. Factors Contributing to the Low Incidence of RH

Currently, there are several factors that may influence the current low occurrence of RH in the Russian Federation.

One such factor is the difficulty in correctly diagnosing this disease. Once inside humans, the RH parasites do not manifest specific clinical features of the disease, particularly during the initial stages; thus, this interferes with the early detection of the infection [34].

Previous experiences have demonstrated that quite often, the RH is detected by chance during the routine clinical and laboratory examinations of patients with respect to various somatic diseases [3]. For example, people harboring the larvae of anisakiosis were detected in the process of laboratory examinations for gastritis and stomach ulcers. Similarly, there was a case of disseminated strongyloidosis in a 52-year-old woman in Volgograd city (Volga River Basin Federal Okrug) as a result of repeated misdiagnoses. She had been treated for a duodenal ulcer for more than 15 years, and no stools had ever been examined [16].

Another way of detecting rare helminthes is through regular medical checkups for specific occupational categories of the population engaged in food processing and restaurant services [2].

One of the peculiarities of the epidemiology of RH in Russia is the low frequency of parasites found in humans in areas with a high prevalence of infected domestic animals. Thus, in the territory of the Central Federal District (Yaroslavl and Bryansk regions), the incidence of fasciolosis among cattle could be as high as 43–100%, while only sporadic cases of the disease were found among the local inhabitants. Another reason could be that in some instances, as in the case of fasciolosis and strongyloidosis in humans, the diagnostic methods were not efficient for particularly low rates of infection [9,35].

Paradoxically, even though the territory of Primorsky is in a close proximity to the endemic areas of Japan, only 16 cases of anisakiosis were reported during the 1990s, as compared with hundreds of cases in Japan [36].

A parasitological examination of 529 specimens of 7 fish species from different water sources in the Khabarovsk territory (2009–2013) revealed a high extensity (11.75 to 100%) and intensity (more than 9 000 larvae/fish) of infection with *N.s.schichobalowi* metacercaria in salmon-like fishes from the mountain tributaries of the Amur river [28,29]. However, the high prevalence of nanophyetosis in local fishes was not related to the high density of parasites in humans [4].

Mixed-infectious helminthes exacerbate the difficulty of detecting RH forms. The presence of one helminthes may mask the existence of other worms in humans. Mixed cases of metorchiosis and opisthorchosis were described in the Siberian and Far East Federal Districts. Difficulties were experienced in the timely diagnosis of pseudamphistomosis in 69 mixed cases of opisthorchosis in the Republic of Tatarstan (Volga River Basin Federal District) during 1989–1994 [4]. Moreover, coproovoscopy and serologic methods failed to accurately define the species-specific affiliation; in this regard, the identification of parasites using DNA diagnostic techniques was deemed necessary [21,22]. An absence or paucity of reported RH cases in the Ural and Siberian Federal Districts (known for their high endemicity of opisthorchosis) could be due to the presence of mixed cases, where *Opistorchis felineus* worms mask the presence of other helminthes like *Mitorchis billis* and *Pseudamphistomum truncatum*. This is because both parasites belong to the family *Opistorchidae* and morphologically resemble *Opistorchis felineus* species [22].

The duration of the incubation period and the longevity of infection of RH forms contribute to problems in the timely detection and reporting of infections [37]. In the case of sparganosis, the incubation period may last from 20 days to 14 months. Moreover, the larvae of *Spirometra* may persist in infected humans for several years before primary manifestations of the disease occur [26]. There are known cases of *Paragonimus* worms that survived for up to 40 years in the affected hosts [26,34,37,38].

An important peculiarity of the epidemiology of strongyloidosis is the ability of parasites to complete their developmental cycle in the body of one host (ability for auto-infection), thus affecting the probability of prolonged infection in organisms [9]. Strongyloidosis, in its intestinal form, may persist asymptomatically for 30 years; however, in the case of decreasing/disappearing cellular immunity in humans, latent infections are able to reactivate [4].

### 3.4. Diversity of the Clinical Manifestations of RH

The clinical manifestations of the RH can vary broadly. In the case of clonorchosis, normally, symptoms of infection are absent or rather mild [39]. Acute disease has not been observed, even in cases of large-scale invasions of the parasite [40]. On the other hand, the severity of the disease was found to be correlated with the excretion rates of helminthes [40]. Clinical manifestations were reported in 5–10% of all cases and basically related to severe liver infections that could have non-specific symptoms such as pain in the upper part of the thoracic cage, meteorism, and fatigue. Prolonged infection is associated with several hepato-biliary diseases like cholecystitis, pyogenic cholangitis, obstructive jaundice, and cholelithiasis [40].

In the case of anisakiosis, the worms can live in humans for several months and cause a very acute reaction to the organism, though without specific symptoms. The absence of specific symptoms in anisakiosis usually results in misdiagnoses in the forms of acute intoxication, gastritis, ulcers, appendicitis, and other gastrointestinal abnormalities. Evidence show that acute cases may develop into a chronic form, mimicking a case of cancer [4,34].

Nevertheless, there are RH forms that demonstrate clinical manifestations during the early stages of infection, as in the case of gastrodiscoidosis (amphistomiosis) symptoms, which are well-defined and related to severe stool disorders [4].

Another peculiarity of RH forms is the presence of different clinical forms of infection, of which local medical personnel may not be aware [12]. In the case of capillariosis, there are several clinical forms of the disease, including the intestinal, pulmonary, and liver, which have different clinical symptoms [26].

In the case of paragonimosis, there are two clinical forms of the disease: classic and larval paragonimosis. Unlike the classic form of the disease, the larval form occurs with a large number of clinical manifestations. However, this fact may complicate the diagnosis of larval paragonimosis, as the abundance of clinical presentations can result in the misdiagnoses of any number of other diseases [41].

Another problem is that some RH forms like metagonimosis may be caused by two species of worms: *Metagonimus yokogavi* (*Katsurada,* 1912) and *Metagonimus katsuradai* (*Isumi,* 1935). Both species are distributed throughout the Primorsky territory. The source of invasion for humans may be fishes from the genus *Cyprinus* (*Linnaeus,* 1758). The parasite density is very high in these fishes and is close to 100%. However, an evolutionary preponderance of parasites in scales and fins (inedible or rarely edible) in these fishes explains the low probability of contraction of the infection in humans, resulting in a low incidence. Disease is reported almost exclusively in indigenous people of the Primorsky territory [12].

### 3.5. Potentially Life-Threatening RH Infection

If left untreated or treated improperly, chronic forms of RH may pose a serious health problem, as these may result in the death of the patient. Thus, in the case of clonorchosis, only 5–10% patients have severe hepatic manifestations with specific symptoms [40]. However, severe, prolonged infections are usually associated with various hepato-biliary diseases like cholecystitis, pyogenic cholangitis, obstructive jaundice, etc. The results of experimental and epidemiological observations are in good agreement with the role of infection in the etiology of one cancer sub-type, cholangiocarcinoma, or cancer of the bile ducts [42].

A few lethal cases of clonorchosis in association with other diseases were reported in the Khabarovsk territory [27]. The results of the postmortem examinations were positive for methorchosis in mixed infection with *Opistorchis felineus* in Western Siberia (Siberian Federal District) [22]. In humans outside Russia, alariosis has led to the deaths of patients in some cases.

In anisakiosis, in the absence of therapeutic intervention, a chronic form of the disease, may result in the death of a patient. The conversion of the disease into its chronic form is facilitated by the remarkable resistance of helminthes to the impacts of environmental conditions [43].

Dioctophymosis is characterized by severe, sometimes lethal complications of the kidney and bladder [44]. Although rare, in cases of a prolonged course of the disease, dicrocoeliosis and fasciolosis may be responsible for biliary cirrhosis of the liver [35]. The brain localization of *Paragonimus westermani* is associated with the probable death of the patient [4].

The development of hyper-infection syndrome, with multi-clinical manifestations in the case of strongyloidosis, may augur the death of a patient. One case of death due to infection was described in 1995 in Moscow city, among six cases of strongyloidosis in children [9,30].

Considering their very low prevalence, the lethality of many RH forms in chronic, untreated cases appears to be high compared with “conventional”, widely distributed helminthes infections like ascaridosis, enterobiosis, etc., thus stressing the importance of highlighting RH as a public health problem in the Russian Federation [4,26].

### 3.6. Epidemiological Peculiarities of RH

There is a paradoxical low frequency of parasites in areas with a very high prevalence of infected wild and domestic animals, fishes, and other sea and river products, as well as contaminated vegetation, water, and soil. Thus, in Russia, the incidence of fasciolosis among cattle can be as high as 100% in certain areas, while only sporadic cases of the disease are detected among the local inhabitants [4].

Although cases of alariosis in humans have not been reported in Russia thus far, *Alaria alata* mesocercaria was found in dogs in Moscow city and in the Moscow, Smolensk, Vladimir, and Kaluga regions. Parasites were found in cats in Moscow and its surrounding regions. In the Khabarovsk territory, the highest infection rate was found amongst domestic cats (46%), and in some places, its incidence was up to 81% [27].

### 3.7. Imported RH Cases in the Russian Federation

Several cases of RH are being imported into the territory of the Russian Federation annually. For example, during 2006–2008, a total of 48 cases of nine species of worms were imported to Russia from 28 foreign countries. Russian citizens were responsible for the importation of the RH in 55% of the total imported cases; the majority of these cases were imported into Moscow city. The rest of the imported cases originated from foreigners [3].

### 3.8. Reporting System

The quality and reliability of reporting systems, with respect to rare helminthes in the Russian Federation, heavily depend on the skill and knowledge of local people with medical expertise, which, currently, are not close to the expected level. Thus, the results of one survey among the personnel of health treatment facilities in the Far East Federal District revealed that only 22% had good knowledge of the epidemiology, diagnosis, clinical manifestations, and treatment of RH [3,4,9].

## 4. Discussion

In Russia, cases of “rare helminthes” in humans have been registered in the territories of six federal districts. There are three distinct groups of infections with respect to the epidemiological and clinical peculiarities, including a total of 15 species of worms. According to the present health information system in Russia, all of these species fall into the category of “other helminthes”, indirectly minimizing their importance for public health and contributing to problems in their early diagnosis and timely treatment; this may result in the occurrence of complicated diseases and, in some cases, in the deaths of patients. Consequently, the health personnel in the majority of health treatment facilities are not familiar with the peculiarities of the epidemiology, clinical manifestations, and treatment of RH cases. Similarly, local populations are not aware of these diseases. Therefore, there is a need to include RH in the training programs for medical staff at all levels of public health institutions, in addition to strengthening health education activities for populations residing in endemic territories.

It should also be kept in mind that the problem of RH may become more pronounced than it is at present. It should be considered as a consequence of global population growth, resulting in limited opportunities to considerably increase the productivity of domestic animals. Therefore, there may be plans to compensate for deficiency in animal proteins by increasing the consumption of sea products. It can be expected that the increased consumption of sea products will result in increased risks of human exposure to the parasites of sea-dwelling species.

In Russia, during the last few years, the consumption of sea products has demonstrated an upward trend.

This trend may be associated with Russians’ ever-increasing appreciation of the cuisine and culinary traditions of Japan, Korea, China, and other countries of Southeast Asia and the Pacific, where many dishes (“sushi”, “sashimi”, “tela”, and others) are based on the use of raw or semi-raw sea fish, shrimps, calamari, etc. Such newly acquired tastes could potentially result in various infections, like anisakiosis and others. For example, popular fishes such as halibut (*Pleuronectes platessa*), dog salmon (*Oncorhynchus keta*), capelin (*Mallotus villiosus*), and herring (*Clupea harengus*) from the Okhotsk Sea are infected with helminthes, with incidences 25% to 100% [24,25].

The potential for fasciolosis in Russia is high due to the high rate of infection in domestic and wild animals. In some regions, the infection rate in domestic animals is as high as 90–100% [11].

Thus, the prevention of new and recurrent helminthiases is an important aspect of public health in Russia and demands the combined efforts of epidemiologists, parasitologists, and hygienists, in association with integrated systems of control to be carried out by personnel involved in agriculture and public health and in the municipalities [1].

## Data Availability

All documents and publications in Russian are available from the Archive and Library of the Martsinovski Institute of Sechenov University, Moscow, Russian Federation.

## Appendix A. Epidemiological Profiles of Non-Rare Helminthes Reported in the Russian Federation

**Distribution (Scale)**	**Helminthes**	**Global Estimation**	**Incidence in Russia**
Global	*Ascaris lumbricoides*	≈800–900 million cases in a total of 153 countries out of 218	24.22 per 100,000 people
	*Trichuris trichiura*	≈500–700 million cases	0.36 per 100,000 people
	*Hymenolepis nana*	Incidence among children >80%	0.13 per 100,000 people
	Enterobius vermicularis	350–500 million cases	153.4 per 100,000 people
Regional	*Opistorchis Felineus*	16,315 (11,273–22,860) cases	Up to 500 per 100,000 people. Basin of Ob’-Irtish River in Siberia, Volga River, Kama River
	*Diphylobothrium latum*	20 million cases	5.38 per 100,000 people in the European part of Russia + Far East
Focal	*Tenia solium*, *Echinococcus granulosis*, *Trichinella* spp., *Toxocara canis*	*Taenia solium* 370,710 (282,937– 478,123); *Echinococcus granulosus* 188,079 (156,848– 1,770,405); *Trichinella* spp. 4472 (2977–5997)	Incidence > 1 per 100,000 people in the European and Asian parts of Russia

## Appendix B. Patterns of Rare Helminthes in Russia vs. the Rest of the world

**District**	**Helminthes**	**In Russia**	**Rest of the World**
Central Federal District	*Dipylidium caninum*	Sporadic cases	Globally endemic
	*Sparganum (Spirometra)*	Sporadic cases	Endemic in Southeast Asia
	*Strongyloides stercoralis*	Sporadic cases, sometimes small-scale outbreaks	Common worldwide in rural communities lacking proper sanitation
	*Fasciola hepatica*	Sporadic cases	Globally endemic
Northwestern Federal District	*Sparganum (Spirometra)*	Sporadic cases	Endemic in Southeast Asia
	*Dioctophyme renale*	Sporadic cases	Globally endemic, including republics of Central Asia in the ex-USSR
	*Strongyloides stercoralis*	Sporadic cases with occasional small-scale outbreaks	Common worldwide in rural communities lacking proper sanitation
Southern Federal District	*Fasciola hepatica*	Sporadic cases only	Globally endemic
	*Strongyloides stercoralis*	In the Central and Northwestern Federal District	Common worldwide in rural communities lacking proper sanitation
	*Gastrodiscoides hominis*	Sporadic cases	Endemic in India, Pakistan, Malaysia, Myanmar, Kazakhstan, Azerbaijan
	*Pseudamphistomum truncatum*	Sporadic cases, occasionally, mixed with *Opisthorchis felineus* found in the basins of Volga and Don Rivers	Sporadic cases
Volga River Basin Federal District	*Capillaria hepatica and Capillaria phillippinensis*	Sporadic cases	Endemic regionally in Southeast Asia
	*Dipylidium caninum*	Sporadic cases	Globally endemic
	*Echinochasmus perfoliatus*	Sporadic cases, mainly in the Astrakhan and Volgograd regions	Sporadic cases
	*Gastrodiscoides hominis*	Sporadic cases	Endemic in India, Pakistan, Malaysia, Myanmar, Kazakhstan, Azerbaijan
	*Fasciola hepatica*	Sporadic cases only	Globally endemic
	*Pseudamphistomum truncatum*	Very rare	Sporadic cases
	*Sparganum (Spirometra)*	Sporadic cases	Endemic in Southeast Asia
	*Trichostrongylus colubriformis*	Sporadic cases	Sporadic cases
	*Strongyloides stercoralis*	Sporadic cases, occasionally small-scale outbreaks	Common worldwide in rural communities lacking proper sanitation
	Mixed cases were found (*Opisthorchis felineus + Pseudamphistomum truncatum*)		
Ural Federal District	Cases of “rare helminths’ in humans not found thus far		
Siberian Federal District	*Metorchis bilis*	Confined to the territory of Western Siberia	Endemic in Manchuria, the Balkan states, Israel, and Spain
	*Opisthorchis felineus*	Endemic in the basin of the Ob’ River	Globally endemic
	*Fasciola hepatica*	Sporadic cases	Globally endemic
	*Pseudamphistomum truncatum*	Cases were found in Tomsk city	Sporadic cases
	Mixed cases are quite common (37,8%)		
Far East Federal District	*Clonorchis sinensis*	Cases are regularly found among local people inhabiting territories in the basin of Amur River	Endemic in China, Japan, Korea, etc.
	*Metagonimus yokogawai*	Endemic among local populations in the Far East of Russia	Endemic in Manchuria, the Balkan states, Israel, and Spain
	*Nanophyetus schichobalowi*	Endemic among local people inhabiting areas in the basin of the Amur River	Endemic in North America, East Asia
	*Paragonimus westermani ichunensis*	Endemic in Far East Federal District, with an incidence of ≈1%	Endemic throughout East, Southwest, and Southeast Asia and South America
	*Anisakis* spp.	Endemic	Endemic all over the world due to the global seafood trade, particularly sregions where dishes are prepared from raw sea fish

## References

[1] Sergiev V.P. (1991). The registered and true prevalence of parasitic diseases. Med. Parazitol..

[2] Sergiev V.P., Uspenskiĭ A.V., Romanenko N.A., Gorokhov V.V., Supriaga V.G., Starkova T.V., Morozov E.N., Chernikova E.A. (2005). “New and recurring” helminthiasis as a potential factor of socioepidemic complications in Russia. Med. Parazitol..

[3] Guzeeva M.V., Guzeeva T.M. (2011). Rare helminthiases. Med. Parazitol..

[4] Lysenko A.Y., Vladimova M.G., Kondrashin A.V., Majori G. (2002). Clinical Parasitology.

[5] Sergiev V.P., Gorokhov V.V., Uspenskiĭ A.V., Romanenko N.A., Maksimov A.A., Moskvin A.S., Lutovinov V.I., Kiselev A.A. (2003). Animal and human cestoda infection (sparganosis). Med. Parazitol..

[6] Uspensky A.V., Gorokhov V.V. (2012). Parasitic Zoonoses.

[7] Skryabin K.I., Shikhobalova N.P., Orlov I.V. (1957). Basics of Nematodology.

[8] Kontrimavichus V.L., Delamure S.L., Boev S.N. (1976). Basics of Nematodology.

[9] Bronshteĭn A.M., Malyshev N.A., Milonova N.G., Aliautdina L.V. (2004). Strongyloidiasis in Moscow region. Med. Parazitol..

[10] Gitsu G.A. (2012). Rare cases of human fascioliasis. Med. Parazitol..

[11] Gorokhov V.V., Molchanov I.A., Maĭsheva-Kolesnikova M.A., Gorokhova E.V. (2011). The fasciolosis epizootic situation in Russia. Med. Parazitol..

[12] Cherkassky B.L. (1994). Infectious and Parasitic Diseases of Man.

[13] Kiselev V.S. (2004). Cutaneous capillariosis. Med. Parazitol..

[14] Sergiev V.P., Uspenskii A.V., Gorokhov V.V., Moskvin A.S., Ivanov V.M., Lomakin V.V., Gorokhova E.V. (2008). Gastrodiscoidosis is a dangerous zoonosis. Med. Parazitol..

[15] Khamidullin R.I., Fomina O.A., Sultanaeva E.G., Khamidullin I.R. (1995). Opisthorchiasis and pseudamphistomiasis on the territory of the middle Volga valley. Med. Parazitol..

[16] Boruk T.F., Plyushcheva G.L., Zelya O.P. (2015). A Local Case of Chronic Strongyloidiasis in the Volgograd Region. Med. Parazitol..

[17] Sergiev V.P., Gorokhov V.V., Romanenko N.A., Moskvin A.S., Romanenko L.N., Migacheva L.D., Vasil’ev D.B., Volichev A.N. (2000). Detection of pentastomes in Russia. Med. Parazitol..

[18] Merekov N.A., Khristianovskii P.I. (2007). Animal helminthisms and their zonal prevalence in the Orenburg Region. Med. Parazitol..

[19] Mordvinov V.A., Yurlova N.I., Ogorodova L.M., Katokhin A.V. (2012). Opisthorchis felineus and Metorchis bilis are the main agents of liver fluke infection of humans in Russia. Parasitol. Int..

[20] Kuznetsova V.G., Naumov V.A., Belov G.F. (2000). Methorchiasis in the residents of Novosibirsk area, Russia. Cytobios.

[21] Brusentsov, Katokhin A.V., Sakharovskaia Z.V., Sazonov A.E., Ogorodova L.M., Fedorova O.S., Kolchanov N.A., Mordvinov V.A. (2010). DNA diagnosis of mixed invasions of Opisthorchis felineus and Metorchis bilis by polymerase chain reaction. Med. Parazitol..

[22] Il’inskikh E.N., Novitskii V.V., Il’inskikh N.N., Lepekhin A.V. (2007). Opisthorchis felineus (Rivolta, 1884) and Metorchis bilis (Braun, 1890) infections in population of some regions of the Ob River Basin. Parazitologiia.

[23] Sergiev V.P., Uspenskii A.V., Sorokina N.P., Syskova T.G., Gorokhov V.V., Romanenko N.A., Molchanov I.A. (2004). Human fascioliasis: Status of the problem. Med. Parazitol..

[24] Ermolenko A.V., Bespozvannykh V.V., Rumyantseva E.E., Voronok V.M. (2015). Pathogens of Human Trematodiases in the Primorye Territory. Med. Parazitol..

[25] Ermolenko A.V., Rumiantseva E.E., Bartkova A.D., Voronok V.M., Poliakova L.F. (2013). Nematodes of humans in the Primorye Territory. Med. Parazitol..

[26] Sergiev V.P., Yushchuk N.D., Vengerov Y.Y., Zavoikin V.D. (2015). Tropical Diseases.

[27] Figurnov V.A., Chertov A.D., Romanenko N.A., Grigorenko A.A., Gavrilov V.A., Soldatkin P.K., Prokhorov P.P., Solozhenkin V.G., Kalinina V.V., Katin I.S. (2002). Clonorchiasis in the Upper Amur region: Biology, epidemiology, clinical presentation. Med. Parazitol..

[28] Dragomeretskaia A.G., Zelia O.P., Trotsenko O.E., Ivanova I.B. (2014). Social bases for the functioning of nanophyetiasis foci in the Amur region. Med. Parazitol..

[29] Dragomeretskaia A.G., Zelia O.P., Trotsenko O.E. (2014). Assessment of Nanophyetus salmincola schikhobalowl (Skrjiabin et Podjiapolskaja, 1931) infestation of salmonlike fishes in the rivers of the Khabarovsk Territory. Med. Parazitol..

[30] Bronshtein A.M., Korablev V.N., Iarotskii L.S. (1992). Intestinal trematodiases (metagonimiasis, nanophyetiasis): Clinico-parasitological research and the first trial of using Azinox in a focus of the lower Amur River valley. Med. Parazitol..

[31] Vitomskova E.A., Dovgalev A.S. (2001). Rates of infection of fishes from Okhotsk sea with human Anisakidae. Med. Parazitol..

[32] Karmanova I.V., Plashkova V.V., Nechaeva O., Gubina V.V. (2002). A case of human anisakiasis in Kamchatka. Med. Parazitol..

[33] Fattakhov R.G., Ushakov A.V., Stepanova T.F., Ianovich V.A., Kopylov P.V. (2012). Epizootiological characteristics of clonorchiasis foci in the Amur River ecosystem in the Jewish autonomic region. Med. Parazitol..

[34] Boireau P. (2014). Multicriteria-Based Ranking for Risk Management of Food-Borne Parasites: Report of a Joint FAO/WHO. Proceedings of the Expert Meeting.

[35] Mas-Coma S. (2005). Epidemiology of fascioliasis in human endemic areas. J. Helminthol..

[36] Gorokhov V.V., Sergiev V.P., Romanenko N.A. (1998). Anisakiasis as a growing ecological and social problem. Med. Parazitol..

[37] Cui J., Lin X.M., Zhang H.W., Xu B.L., Wang Z.Q. (2011). Sparganosis, Henan Province, central China. Emerg. Infect. Dis..

[38] Sripa B., Kaewkes S., Intapan P.M., Maleewong W., Brindley P.J. (2010). Food-borne trematodiases in Southeast Asia epidemiology, pathology, clinical manifestation and control. Adv. Parasitol..

[39] Chelomina G.N. (2014). Current treatments for clonorchiasis. Med. Parazitol..

[40] Dyk L.M., Posokhov P.S., Dobrykh V.A., Markina L.G., Kozyreva T.G. (1997). Experience with a clinical parasitological examination in a clonorchiasis focus in the Amur River region. Med. Parazitol..

[41] Ermilov V.V., Smirnov A.V., Snigur G.L., Dudin R.S., Popov S.S. (2018). Pulmonary larval paragonimiasis mimicking lung cancer. Arkhiv Patol..

[42] Shin H.R., Oh J.K., Masuyer E., Curado M.P., Bouvard V., Fang Y., Wiangnon S., Sripa B., Hong S.T. (2010). Comparison of incidence of intrahepatic and extrahepatic cholangiocarcinoma--focus on East and South-Eastern Asia. Asian Pac. J. Cancer Prev..

[43] Lymbery A.J., Cheah F.Y. (2007). Anisakid Nematodes and Anisakiasis. Food-Borne Parasitic Zoonoses.

[44] Vladimova M.G., Lysenko A., Gorbunova Iu P., Avdiukhina T.I., Konstantinova T.N., Romanenko L.N. (2002). A case of dioctophymosis (Dioctophyme renale) in a girl from Arkhangelsk. Med. Parazitol..

